# Laser Synthesis and Photocatalytic Properties of Bismuth Oxyhalides Nanoparticles

**DOI:** 10.3390/nano14241995

**Published:** 2024-12-12

**Authors:** Vyacheslav E. Korepanov, Olesia A. Reutova, Tamara S. Kharlamova, Olga V. Vodyankina, Sergei A. Kulinich, Valery A. Svetlichnyi

**Affiliations:** 1 Laboratory of Advanced Materials and Technology, Tomsk State University, Tomsk 634050, Russia; 2 Department of Physical and Colloid Chemistry, Faculty of Chemistry, Tomsk State University, Tomsk 634050, Russia; 3 Research Institute of Science and Technology, Tokai University, Hiratsuka, Kanagawa 259-1292, Japan

**Keywords:** bismuth oxyhalides, photocatalyst, nanoparticles, laser synthesis, decomposition of organic pollutants, selective oxidation, 5-hydroxymethylfurfural

## Abstract

Photocatalysis offers a powerful approach for water purification from toxic organics, hydrogen production, biosolids processing, and the conversion of CO_2_ into useful products. Further advancements in photocatalytic technologies depend on the development of novel, highly efficient catalysts and optimized synthesis methods. This study aimed to develop a laser synthesis technique for bismuth oxyhalide nanoparticles (NPs) as efficient and multifunctional photocatalysts. Laser ablation of a Bi target in a solution containing halogen salt precursors, followed by laser plasma treatment of the resulting colloid, yielded crystalline bismuth oxyhalides (Bi_x_O_y_X_z_, where X = Cl, Br, or I) NPs without the need for additional annealing. The composition, structure, morphology, and optical properties of the synthesized Bi_x_O_y_X_z_ (X = Cl, Br, I) NPs were characterized using XRD analysis, electron microscopy, Raman spectroscopy, and UV-Vis spectroscopy. The effect of the halogen on the photocatalytic activity of the double oxides was investigated. The materials exhibited high photocatalytic activity in the degradation of persistent model pollutants like Rhodamine B, tetracycline, and phenol. Furthermore, the Bi_x_O_y_X_z_ NPs demonstrated good efficiency and high yield in the selective oxidation of 5-hydroxymethylfurfural (5-HMF) to 2,5-furandicarboxylic acid (FDCA). The obtained results highlight the promising potential of this laser synthesis approach for producing high-performance bismuth oxyhalide photocatalysts.

## 1. Introduction

Photocatalysis research remains a highly dynamic field due to its significant environmental impact and broad applications. These include the degradation of hazardous organic [1], inorganic [2], and biological pollutants [3]; hydrogen production [4,5]; the conversion of biomass waste into high-value products [6,7,8]; and the reduction of CO_2_ to valuable fuels like methane, methanol, and ethanol [9,10,11]. Future advancements in photocatalytic technologies rely on the creation of highly efficient and stable catalysts, necessitating improved methods for synthesizing nanomaterials with tailored structures and properties, and a deeper understanding of the underlying photocatalytic mechanisms.

Along with traditional chemical methods for synthesizing nanomaterials, such as the sol-gel technique, in recent years, various physical approaches have been increasingly applied, such as high-energy laser synthesis [12], which is often based on the pulsed laser ablation (PLA) method [13,14]. PLA in liquid is a green technology approach that offers several advantages, including a minimal use of solvents and precursors, the versatility and adaptability of the method, and the ability to prepare a wide variety of nanostructures. By altering the target material, reaction medium (solvent, precursors), laser exposure parameters (power density, pulse duration, and radiation wavelength), and other experimental conditions, it is possible to control the composition and structure of the obtained materials over a wide range [15]. High-energy non-equilibrium conditions generated in laser synthesis enable the creation of efficient photocatalysts with unique optical properties, active surfaces, and defective structures. Advances in productivity, automation, and cost reduction allow PLA to be used not only for the synthesis of model materials for proof-of-concept studies, but also for the production of catalysts for laboratory and pilot applications [16,17]. Even greater success is expected from combining different techniques based on their features and advantages, especially when preparing complex composites, multiphase nanomaterials, supported catalysts, etc. [18].

Among a wide range of known oxide semiconductor photocatalysts, both single-phase and complex bismuth oxides have been reported [19]. When compared to the known classical catalysts based on TiO_2_ and ZnO, these materials often have a smaller bandgap, which permits the more efficient use of the visible part of the spectrum. Many complex bismuth oxides with various metals (V, W, Fe, Mo, Ba, etc.) [20,21] and nonmetals (Si, P) [22,23] and heterostructures based on them [24] were also used in photocatalysis. The layered structure of some complex bismuth oxides is known to lead to better separation of photogenerated charge carriers, which can contribute to increased photocatalytic activity of such materials [25].

Bismuth oxyhalides Bi_x_O_y_X_z_ (X = Cl, Br, I) are considered to be among the most efficient Bi-based oxide photocatalysts. In addition to other properties, they have demonstrated efficiency in the decomposition of various organic pollutants [26], including pharmaceutical substances [27], as well as CO_2_ reduction [28]. The availability of bismuth oxyhalides with different stoichiometric Bi/X ratios (e.g., BiOI, Bi_4_O_5_Br_2_), as well as the existence of ternary oxides with two halogen elements simultaneously [27] and various heterostructures on their basis [29,30] permits the control of the spectral properties and activity of such catalysts. At present, the most common method for the preparation of bismuth oxyhalides is hydro/solvothermal synthesis [31,32]. Such nanomaterials can also be obtained, for example, by co-precipitation [26,28], and microwave-assisted solvothermal method [33]. In light of the above, it can be assumed that using laser methods for the synthesis of Bi_x_O_y_X_z_ nanostructures will provide new opportunities to control their structure and properties, as well as to create novel composite catalysts.

In this study, we developed a novel approach for the preparation of Bi_x_O_y_X_z_ nanoparticles (NPs), which is entirely based on high-energy pulsed laser action in a liquid phase. Specifically, we initially used laser ablation of a metallic bismuth (Bi) target in an aqueous potassium salt solution of the corresponding halogen (X = Cl, Br, I). This was then followed by laser processing of the resulting colloid with tightly focused radiation from the same laser. We also analyzed the morphology and structure of the newly prepared nanostructures, their optical properties, and finally tested their photocatalytic activity in the decomposition of known persistent organic compounds and the selective oxidation of 5-hydroxymethylfurfural (5-HMF).

## 2. Materials and Methods

### 2.1. Laser Synthesis of Bismuth Oxyhalides

The procedure developed and used for laser synthesis of bismuth oxyhalides in this work had two stages. At stage I, a colloidal solution of NPs was obtained by pulsed laser ablation (PLA) of bismuth in a medium with halogen ions X^−^ (X = Cl, Br, or I) as precursors. Ablation was performed by applying focused main harmonic radiation of a Nd:YAG laser (LS-2031M, from Lotis TII, Belarus, Minsk) with a wavelength of 1064 nm, pulse duration of 7 ns, pulse energy of 150 mJ, and pulse repetition rate of 20 Hz to a target of metallic Bi (99.5% purity) in aqueous solutions of three different potassium halides KX (X = Cl, Br, and I). The concentration of KX salt was chosen in excess of bismuth so that the Bi/X molar ratio was ~1:20. Since metallic Bi has rather low melting (545 K) and vaporization (1837 K) temperatures and heat of vaporization (170 kJ/mol), this determines its low ablation threshold. Therefore, colloidal NPs were laser-produced under relatively mild conditions, i.e., at a low radiation power density on the target surface of only about ~150 MW/cm^2^. As a result, no sufficiently effective interaction between the Bi species ablated from the target surface and X^−^ ions in liquid medium occurred during this stage. Therefore, at stage II, the obtained colloidal solution was further treated with irradiation from the same Nd:YAG laser which was hard-focused (with a lens with F = 50 mm) into the center of the reactor under constant agitation with a magnetic stirrer. Stage II lasted 2 h, with the irradiation power density at the focal point reaching several GW/cm^2^, thus exceeding the breakdown threshold of the medium and resulting in plasma glow observed. Such a high-energy plasma mode of laser treatment of colloidal solution promoted effective interaction of particles with their medium leading to formation of bismuth oxyhalides. As a result of laser treatment in plasma mode (stage II), the color of the solution changed from dark-brown to white or yellowish (depending on the halogen X used) leading eventually to the formation of flakes that precipitated after the stirring was stopped. As the last step, the obtained dispersions were centrifuged using a FC5515R centrifuge (Ohaus, Albstadt, Germany) at 12,000 rpm with cooling to 5 °C. The resulting precipitate was rinsed three times with distilled water and then air dried at 60 °C. The procedure was repeated several times until the amount of powder required for further experiments was obtained. Hereafter, the prepared samples are labeled as Bi_x_O_y_Cl_z_, Bi_x_O_y_Br_z_ and Bi_x_O_y_I_z_.

### 2.2. Sample Characterization

The morphology of the prepared NPs was investigated by transmission (TEM) and scanning (SEM) electron microscopy methods using SM12 (Philips, Amsterdam, The Netherlands) and VEGA 3 SBH (Tescan, Brno, Czech Republic) microscopes, respectively. The energy-dispersive X-ray spectroscopy (EDS) system AztecLive Lite Xplore 30 (Oxford Instruments, Abingdon, UK) was used to evaluate the chemical composition of NPs during SEM studies. The specific surface area (S_BET_) of the catalysts was determined using the BET method from the low-temperature N_2_ adsorption isotherms at 77 K obtained using a TriStar II 3020 analyzer (Micromeritics, Norcross, GA, USA). Each sample was preliminarily degassed in vacuum at 25 °C for 6 h.

The crystal structure of NPs was studied by X-ray diffraction (XRD) using an XRD-7000 diffractometer (Shimadzu, Japan) with the CuKα radiation (*λ* = 1.5418 Å) in the range of 2θ angles from 5° to 90°. After collecting XRD data, the crystal structure of samples was identified using the PDF-4+ powder database (International Center for Diffraction Data, USA) and the PowderCell 2.4 software (Informer Tech-nologies, Inc., Los Angeles, CA, USA).

Raman shift spectra were measured on an InVia Basic confocal Raman spectrometer (Renishaw, New Mills, UK) under excitation from a semiconductor laser (λ = 785 nm) in the range of 90–1200 cm^−1^ with a spectral resolution of 2 cm^−1^. Optical properties of the powders in the UV-Vis range were investigated on a Cary 100SCAN spectrophotometer (Varian, Australia) by means of diffuse reflectance spectroscopy (DRS) using a DRA-CA-30I based integrating sphere (Labsphere, North Sutton, NH, USA). The powders were mixed with MgO at a ratio of 1:10 for correct measurements, while the MgO powder was also used as a reference. Using the Kubelka–Munk transformation, DRS spectra were converted into absorption spectra, from which the optical bandgap width *E*_g_ of semiconductor NPs was estimated by the Tauc method according to formula (1):

(*αhν*)^1/n^ = *A*(*hν* − *E*_g_), (1)

where *α* is the absorption coefficient, *hν* is the photon energy, *A* is a constant independent of energy, and coefficient *n* = 1/2 for direct and *n* = 2 for indirect zone transition [34].

### 2.3. Photocatalytic Studies

The photocatalytic properties were investigated in the decomposition reactions of two organic molecules, Rhodamine B (Rh B) as a known persistent dye and phenol (Phen) as a model pollutant, as well as antibiotic tetracycline (TC). They were also tested in the selective oxidation reaction of 5-hydroxymethylfurfural (5-HMF) when exposed to 375 nm soft UV light-emitting diode (LED) radiation with FWHM = 10 nm. The total LED optical power was measured by a semiconductor detector PD300UV (Ophir, Jerusalem, Israel) and was 50 mW. For photodegradation of Rh B (5 μM), Phen (50 μM) and TC (50 μM), 15 mg of catalyst was used in a 30 mL volume. Before irradiation, the solutions were incubated for 1 h in the dark to establish an adsorption–desorption equilibrium. A dark stage was performed in the system prior to irradiation. During the experiments the dispersion with NPs and organic compound was stirred with a magnetic stirrer at a speed of 600 rpm. Photodegradation of organic molecules was evaluated by the change of spectral characteristics of solutions for the case of first-order reaction kinetics. The concentration of Rh B and TC was determined from their absorption spectra using a Cary 100SCAN spectrophotometer (Varian, Mulgrave, Australia), while the concentration of Phen was determined from fluorescence spectra using a RF-5301 PC spectrofluorophotometer (Shimadzu, Kyoto, Japan). The reaction rate constant *k* was determined from the slope tangent from formula (2):

ln(*C*_0_/*C*) = *kt*, (2)

where *C*_0_ is the initial concentration, *C* is the current concentration of Rh B or Phen, and *t* is the reaction time.

Selective photooxidation of aqueous 5-HMF (10 mM) was carried out in aqueous solution of Na_2_CO_3_ (40 mM) as a weakly alkaline agent (pH 9). The total volume of the solution was 100 mL, and the catalyst mass was fixed as 100 mg (1 g/L). The total optical power of the LEDs emission was 2 W, and the catalytic processes were carried out at room temperature and atmospheric pressure, with air blown through the solution at a rate of 60 mL/min, while the solutions were stirred at a rate of 600 rpm. Similar to Rh B, the reaction mixture with 5-HMF was first kept in the dark for 1 h. The reaction products were analyzed by high-performance liquid chromatography (HPLC), by using a Prominence-i LC-2030C chromatograph (Shimadzu, Kyoto, Japan). The methodology for the analysis of 5-HMF photoproducts was described in greater detail elsewhere [35].

## 3. Results and Discussion

### 3.1. Morphology, Composition, and Structure of Samples

**Figure 1:**
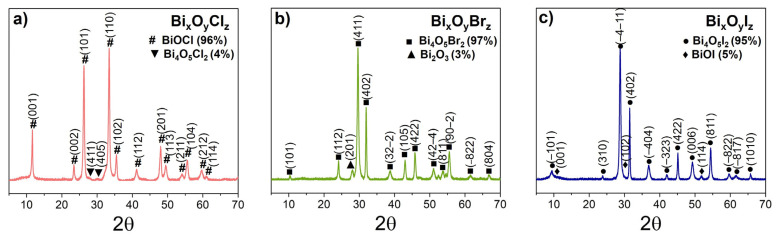
XRD patterns of Bi_x_O_y_Cl_z_ (**a**), Bi_x_O_y_Br_z_ (**b**), and Bi_x_O_y_I_z_ (**c**) powders.

Figure 1 presents XRD patters of the prepared powders. The sample obtained by PLA in the presence of KCl is seen to be by 96% composed of the tetragonal phase BiOCl (PDF4 #01-083-7690, space group *P4/nmm*), while those produced in presence of KBr and KI demonstrate the monoclinic phases Bi_4_O_5_Br_2_ (97%, PDF4 #04-025-6520, space group *P21*) and Bi_4_O_5_I_2_ (95 %, PDF4 #04-012-1474, space group *P21*) as their main phases. In addition to the main phase, a small amount of tetragonal oxide β-Bi_2_O_3_ (PDF4 #04-007-1443, space group $P\bar{4}2_{1}c$) was also detected in sample Bi_x_O_y_Br_z_, whereas samples Bi_x_O_y_I_z_ and Bi_x_O_y_Cl_z_ demonstrated phase BiOI (PDF4 #01-085-4009, space group *P4/nmm*), and phase Bi_4_O_5_Cl_2_ (PDF4 #00-041-0658, space group *Pnma*). The average crystallite size for the main BiOCl, Bi_4_O_5_Br_2_ and Bi_4_O_5_I_2_ phases in the samples was found to be ~35 nm. One can assume that under laser conditions used, as layers of Bi_2_O_3_ oxide are formed during oxidation of metallic Bi, as a result of incorporation of halide ions into it, a predominantly layered BiOX structure is formed. Such a structure is believed to be rather stable in the case of chloride ions (whose radius is 0.181 nm) [36]. In the case of bromide- and iodide ions, due to their larger ion radii (0.196 and 0.219 nm for Br^−^ and I^−^ ions, respectively) [37], it is less stable under laser treatment conditions (high local temperatures). As a result, this leads to the formation of the more stable, in this case, structures Bi_4_O_5_Br_2_ and Bi_4_O_5_I_2_. Similar transitions caused by thermal treatment have been described earlier, for example, for Bi_x_O_y_I [38] and Bi_x_O_y_Br [39]. To form particles of different stoichiometry (different Bi/X ratio), it is planned to change the conditions of laser treatment and the composition of the reaction medium in further studies, as was shown in work [40] for the chemical synthesis of Bi_x_O_y_Br_z_ particles.

Thus, XRD analysis demonstrates that laser treatment in plasma mode of colloidal Bi NPs in the presence of halide ions as precursors permits to produce crystalline NPs of bismuth oxyhalides with rather high yield. This advantageously distinguishes the approach realized in this study from the laser synthesis of bismuth silicates based on laser treatment of a mixture of colloidal Bi and Si NPs produced by PLA [41], where the formation of crystalline particles of complex oxides required additional annealing of powders at temperatures of 400–600 °C [42]. At the same time, crystalline BiVO_4_ nanoparticles without additional annealing were reported by others [43] after the irradiation of bismuth oxide microparticles in a NH_4_VO_3_ solution.

**Figure 2:**
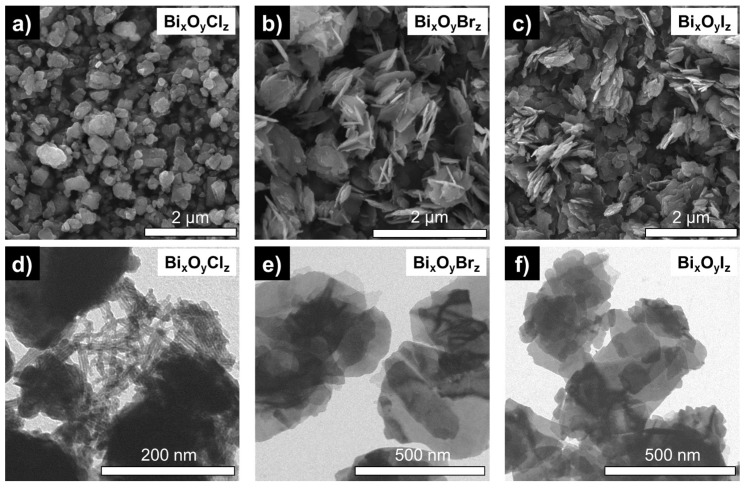
SEM (**a**–**c**) and TEM (**d**–**f**) images of Bi_x_O_y_X_z_ powders.

The morphology of produced Bi_x_O_y_X_z_ powders is presented as SEM images in Figure 2a–c. The Bi_x_O_y_Br_z_ and Bi_x_O_y_I_z_ powders are seen in panels (b,c) to consist of thin plates with lateral dimensions of 0.5–1 μm and 300–600 nm, respectively, with their plate thickness reaching up to several tens of nanometers. In contrast, the Bi_x_O_y_Cl_z_ NPs are seen in panel (a) to be more faceted, with sizes ranging within 100–400 nm. This morphology correlates well with the SEM data previously reported for tetragonal BiOCl and monoclinic phases of Bi_4_O_5_I_2_ [19]. To better visualize the layered structure of NPs, TEM images were obtained. The micro-images presented in Figure 2d–f show that the morphology of particles in samples Bi_x_O_y_Br_z_ and Bi_x_O_y_I_z_ is similar as they predominantly consist of thin plates. The morphology of particles in sample Bi_x_O_y_Cl_z_ is significantly different. The sample is seen in panel (d) to be mainly represented by large bulk particles, which can be attributed to BiOCl, with a small number of small lamellar/rod-like structures.

The electron microscopy data were found to correlate with the measured specific surface area of the samples, as its values increased in the Cl–Br–I halogen series, being 8.8, 12.4, and 14.5 m^2^/g, respectively.

### 3.2. Raman and UV-Vis Spectra, Bandgap of Produced Nanostructures

**Figure 3:**
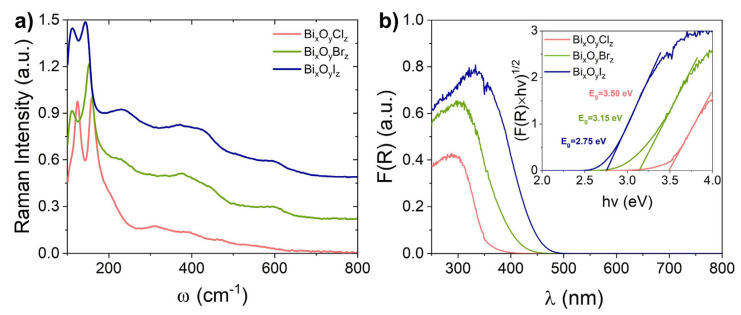
Raman (**a**) and UV-Vis (**b**) spectra of prepared samples; the inset illustrates estimation of the bandgap width of semiconductor NPs by the Tauc method.

The Raman shift spectra of the synthesized powders in the range of 100–800 cm^−1^ are presented in Figure 3a. It is known that part of the intense bands of the Raman spectrum of bismuth oxyhalides related to Bi–Bi vibrations lies in the region below 100 cm^−1^ [44]. In the present spectra measured in the range > 100 cm^−1^, only one of the bands in the low-frequency region is observed, which can be attributed to the *A*_1g_ internal Bi–Br stretching mode [45]. For samples with bromine and iodine, which were shown to have a predominantly monoclinic structure, this band has a maximum at 112 cm^−1^, while for tetragonal sample BiOCl it has a maximum at 124 cm^−1^. Also in the spectra, one can identify the Bi-X libration displacement mode at ~150 cm^−1^, whose position was observed to shift predictably to the low-frequency region with increasing the mass of halogen atom from 159 cm^−1^ to 152 cm^−1^ and to 143 cm^−1^ for samples Bi_x_O_y_Cl_z_, Bi_x_O_y_Br_z_ and Bi_x_O_y_I_z_, respectively. Several broad bands observed in the 200–700 cm^−1^ region can be attributed to various types of O–Bi–O vibrations and overtones of lower frequency vibrations.

Figure 3b shows the UV-Vis spectra of the samples presented in F(R) coordinates obtained by transforming their DRS spectra using the Kubelka–Munk function. As inset, the results of estimating samples’ bandgap width (*E*_g_) by the Tauc method for the case of indirect bandgap optical transitions are shown. It is known from the literature that tetragonal bismuth oxyhalides BiOCl, BiOBr and BiOI exhibit indirect gaps [46]. Monoclinic Bi_4_O_5_Br_2_ [47] and Bi_4_O_5_I_2_ [48] bismuth oxyhalides are also known to belong to indirect bandgap semiconductors. The obtained spectra and the obtained values of optical bandgap width correlate with the known data for the corresponding materials. The Bi_x_O_y_Cl_z_ sample, which was found to have mainly the tetragonal BiOCl phase, is seen in Figure 3b to absorb in the region below 400 nm and has the largest bandgap of all the three samples, 3.5 eV [49]. The absorption of samples Bi_x_O_y_Br_z_ and Bi_x_O_y_I_z_ (which are based on the monoclinic phases Bi_4_O_5_Br_2_ and Bi_4_O_5_I_2_) is seen to extend into the visible region up to 450 and 500 nm, and their estimated bandgap widths are 3.15 eV [50] and 2.75 eV [51], respectively.

### 3.3. Photocatalytic Activity

#### 3.3.1. Rhodamine B

**Figure 4:**
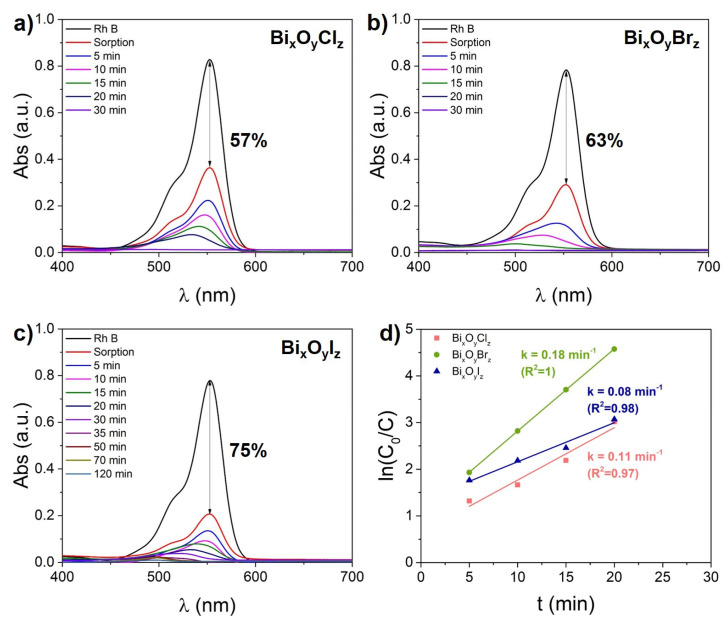
Photocatalytic degradation of Rh B: UV-Vis absorption spectra (**a**–**c**), kinetic curves (**d**) with Bi_x_O_y_X_z_ powder.

Figure 4 presents the results of the photocatalytic decomposition of Rh B by the three materials. As one can see from the absorption spectra in Figure 4a–c, the ionic dye (chloride) is strongly sorbed on the surface of the catalyst NPs. The sorption of Rh B by Bi_x_O_y_X_z_ NPs is seen to increase in the Cl-Br-I halogen series (see red spectra in Figure 4a–c). Similar results on the strong sorption of Rh B on BiOCl particles were observed by Ahmed et al. [52]. As noted in the literature [53], the good sorption properties of the photocatalyst can also be an additional advantage for selective separation and purification of aqueous media from various pollutants.

When analyzing the dynamics of changes in the intensity and shape of absorption spectra during photocatalytic degradation of Rh B, one can notice that the processes of N-diethylated and distortion of the aromatic structure of the dye occur simultaneously and with close efficiency. The N-diethylatation is manifested in the hypsochromic shift of the absorption band to the region of 495 nm, where rhodamine 110 (N-diethylated analog of Rh B) absorbs [54]. At the same time, Rh 110 also continues to degrade upon further irradiation. Figure 4d shows the kinetic curves of photocatalytic degradation of Rh B which are based on evaluation of the decrease in absorbance at a wavelength of 553 nm. It is clearly seen that sample Bi_x_O_y_Br_z_ exhibits the highest photocatalytic efficiency: after as long as 25 min of irradiation, its Rh B solution was completely decolorized and no bands of either Rh B or Rh 110 were observed in the spectrum (Figure 4b). The complete decolorization of the dye solution with catalyst Bi_x_O_y_Cl_z_ is seen in Figure 4a to occur after 30 min. In contrast, in presence of catalyst Bi_x_O_y_I_z_, degradation of the original Rh B (absorption band at 553 nm) occurred in 40 min, while complete decolorization of the solution (absorption band of Rh 110 at 495 nm) was observed only after 120 min (Figure 4c).

**Figure 5:**
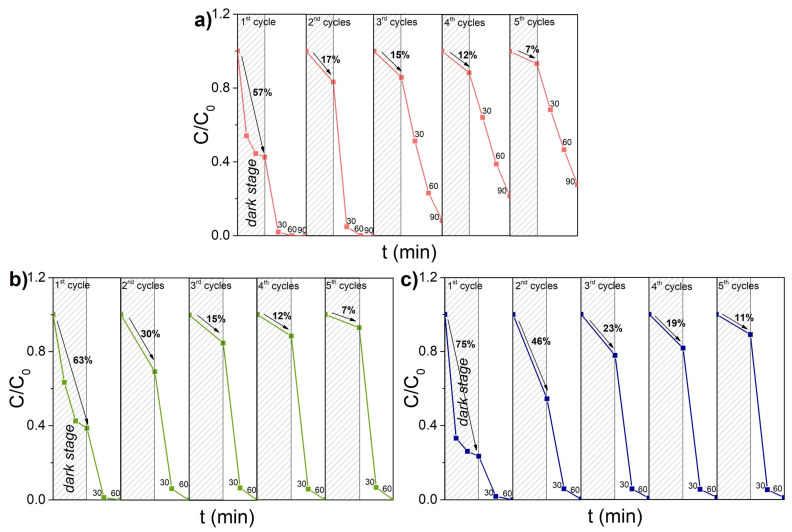
Cyclic stability curves of samples Bi_x_O_y_Cl_z_ (**a**), Bi_x_O_y_Br_z_ (**b**), and Bi_x_O_y_I_z_ (**c**) as they decompose Rh B under irradiation with λ = 375 nm. The shaded area corresponds to the dark sorption before each irradiation cycle.

The stability of the prepared Bi_x_O_y_X_z_ photocatalysts in the N-deethylation process was evaluated during Rh B decomposition under irradiation by LED with a wavelength of 375 nm (Figure 5). After each cycle of photocatalytic tests, a concentrated solution of Rh B dye was added to the reactor. As one can see in Figure 5a, the efficiency of sample Bi_x_O_y_Cl_z_ decreased with each cycle, while samples Bi_x_O_y_Br_z_ (b) and Bi_x_O_y_I_z_ (c) worked steadily for five cycles without any decrease in their efficiency (Figure 5b,c). For all the materials, a decrease in dark sorption during cycling tests can be observed, which can be related to the sorption of decomposition products on the surface of their particles.

#### 3.3.2. Tetracycline

**Figure 6:**
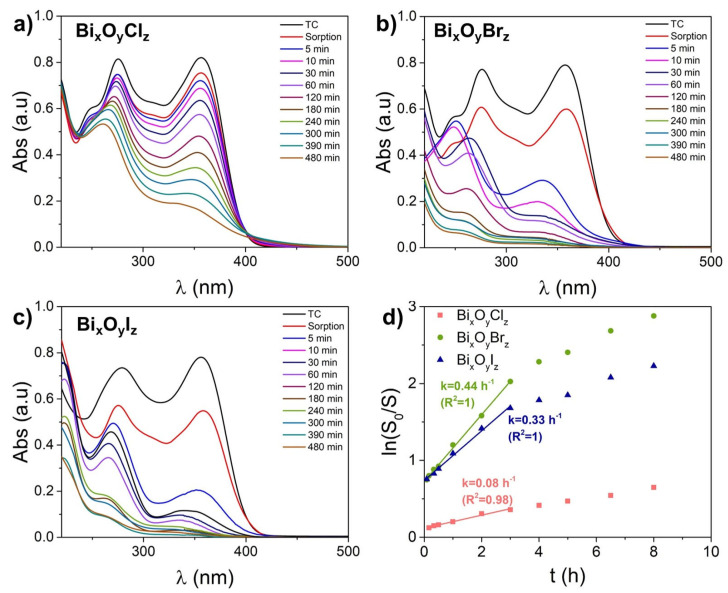
Photocatalytic degradation of tetracycline: UV-Vis absorption spectra (**a**–**c**), kinetic curves (**d**) with Bi_x_O_y_X_z_ powder.

Figure 6 shows the results of the photocatalytic decomposition of TC molecules. The dark sorption of TC hydrochloride molecules on Bi_x_O_y_X_z_ NPs is lower than that of Rh B, but it remains significant (up to 30%) and tends to increase within the Cl-Br-I halogen series. The framework of the TC molecule consists of four six-membered rings which form its absorption spectrum in the region of 250–400 nm (Figure 6a–c) [55]. In absence of catalysts, the photolysis of this antibiotic was reported to occur under hard UV irradiation by a mercury lamp with a wavelength of 254 nm [55]. In the present study, under irradiation with low power soft UV radiation (λ = 375 nm) for 8 h, changes observed in the spectrum around 360 nm were no more than 5% [56]. The TC photodegradation efficiency was evaluated not at an individual wavelength, but by the integral absorption decrease in the whole range of 250–450 nm. The photodegradation rate constant was determined from a linear plot from kinetic curves, as shown in Figure 6d. After irradiation for several hours, the course of the kinetic curves changed, indicating a change in the photodegradation mechanisms. The processes of photocatalytic decomposition of the structure of TC under irradiation with a Xe lamp with 300 W and a 420 nm cut-off filter (420 nm < *λ* < 780 nm) in the presence of a TiO_2_-based composite catalyst were discussed in detail elsewhere [57]. A more detailed analysis of the TC photodegradation mechanisms in the presence of our catalysts will be carried out in the future. At this point, it can be seen from the absorption spectra that the samples Bi_x_O_y_Br_z_ and Bi_x_O_y_I_z_ degraded TC the most efficiently. At the same time, the shape of their absorption spectra over time is seen in Figure 5b,c to change differently, which implies some difference in mechanisms and photodegradation products in presence of these two catalysts. For example, the spectra of sample Bi_x_O_y_Br_z_ demonstrate a hypsochromic shift in the long-wavelength absorption band of TC (Figure 6b).

In parallel, sample Bi_x_O_y_Cl_z_ is seen in Figure 6a to show the lowest efficiency in TC decomposition. It should be noted that LED radiation used (λ = 375 nm) is near the maximum of the long-wavelength absorption band of TC, and therefore, under this irradiation the photocatalyst interacts with antibiotic molecules which are mainly in their excited state. Additionally, since TC itself effectively absorbs this type of irradiation, this can strongly influence the absorption and photocatalytic activity of the Bi_x_O_y_Cl_z_ NPs which absorb weakly in this region.

#### 3.3.3. Phenol

**Figure 7:**
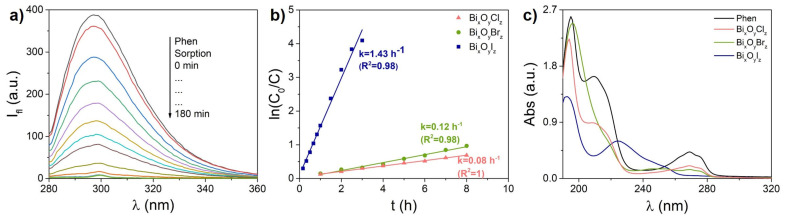
Photocatalytic degradation of phenol in presence of Bi_x_O_y_X_z_ powders: fluorescence spectra for sample Bi_x_O_y_I_z_ (**a**), kinetic curves (**b**) and UV-Vis absorption spectra of initial phenol solution and those after 8 h of degradation in presence of Bi_x_O_y_X_z_ powders (**c**).

Figure 7 presents the results of the photocatalytic degradation of phenol, where Figure 7a shows the fluorescence spectra of phenol during sorption and photocatalysis for the sample Bi_x_O_y_I_z_. In contrast to Rh B, the sorption of the neutral Phen molecule on all catalysts is negligible, being about 5%. It is also worth noting that Phen itself does not absorb the LED radiation used in our experiments (λ = 375 nm), and hence the catalytic NPs could only interact with unexcited analyte molecules. Kinetic curves of photocatalytic degradation of Phen were plotted based on corresponding fluorescence data (Figure 7b). It is clearly seen that the best results were demonstrated by the sample Bi_x_O_y_I_z_ which completely degraded the initial Phen molecules in less than 3 h. The rate constant for the sample Bi_x_O_y_I_z_ was calculated to be three times higher than for the previously reported heterostructure Bi_2_(CO_3_)O_2_/Bi_12_SiO_20_ [58], and ten times higher than for the highly defective dark titanium dioxide [59], which was studied earlier under the same conditions.

Figure 7c compares the absorption spectra of Phen solution before and after photocatalytic degradation by the three samples, for as long as 8 h. Phenol is known to have a long transformation chain as a result of interactions with active particles and radicals during photocatalysis. In this regard, the possible photoproducts formed by the interaction of Phen with reactive oxygen species during photolysis and photocatalysis can be found in works [60,61]. The absorption spectra of a number of compounds that are known intermediate products of Phen photodegradation at neutral pH were reported in [62,63]. Both direct photolysis of Phen under UV irradiation [61,64] and photocatalysis in the presence of TiO_2_ [59,62,63] were shown to give rise to products that absorb in a wide range, so that their absorption spectra overlap with the spectrum of the initial molecule. Figure 7c shows that, in the present work, no formation of hydroxydiphenyls and hydroquinone that absorb in a spectral region longer than that of Phen (>280 nm) was observed. Additionally, the decrease in Phen fluorescence intensity was found to correlate with the decrease in absorbance at the absorption band maximum, implying that photocatalysis did not produce pyrocatechol and resorcinol, which absorb in the region around 275 nm. From the increase in absorption in the region at around 245 nm, one can conclude that the main intermediate containing a benzene ring was p-benzoquinone. The other possible products absorb in a shorter wavelength region, thus indicating that the benzene ring was broken.

#### 3.3.4. 5-Hydroxymethylfurfural

**Figure 8:**
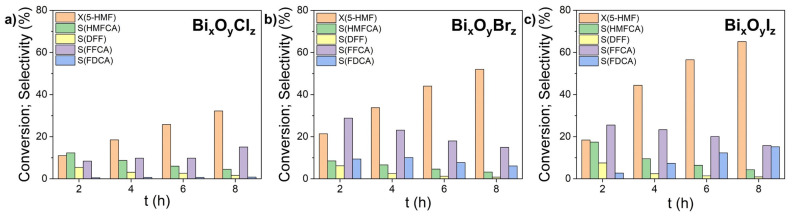
Photocatalytic oxidation of HMF in presence of samples: Bi_x_O_y_Cl_z_ (**a**), Bi_x_O_y_Br_z_ (**b**), and Bi_x_O_y_I_z_ (**c**).

The results of the photocatalytic oxidation of 5-HMF are presented in Figure 8. The reaction scheme and photooxidation products of 5-hydroxymethylfurfural are well enough studied and can be found elsewhere [65]. Having a furan ring, hydroxyl and formyl groups in its structure, 5-HMF is a universal platform for the synthesis of a number of demanded industrial intermediates. As a first step, the selective oxidation of 5-HMF is known to yield 2,5-diformylfuran (DFF) or 5-hydroxymethyl-2-furancarboxylic acid (HMFCA). Upon further oxidation of DFF and HMFCA, 5-formyl-2-furancarboxylic acid (FFCA) and 2,5-furandicarboxylic acid (FDCA) are produced sequentially. Most previous studies on the photocatalytic oxidation of 5-HMF reported on DFF as a product [66]. The latter intermediate, DFF, is used in the preparation of pharmaceuticals, furan-based biopolymers, fungicides, etc. However, FDCA, being a result of deeper oxidation of 5-HMF, is considered to be the most promising product, since it can be used as a basis for the synthesis of a number of polymers, such as polyethylene furanoate (PEF).

Similar to the case of photocatalytic decomposition of phenol, 5-HMF cannot be excited by LED irradiation with λ = 375 nm because it absorbs in the spectral region below 300 nm [67]. The lowest activity in the oxidation of 5-HMF was demonstrated by sample Bi_x_O_y_Cl_z_ (Figure 8a), which also weakly absorbs radiation with this wavelength. In the first oxidation step in the presence of sample Bi_x_O_y_Cl_z_ (as well as for the other two catalysts), the HMFCA formation channel was the most efficient. This product was then oxidized further to FFCA which remained the main product for the Cl-containing catalyst. After 8 h of irradiation, the overall conversion of 5-HMF was 32%, with the FFCA yield being 15% (Figure 8a).

When the sample Bi_x_O_y_Br_z_ was used under similar conditions, the conversion of 5-HMF reached 52%, with FFCA and FDCA being the main products. However, the maximum selectivity (28% for FFCA and 10% for FDCA) was achieved at the initial stage of catalysis. Thereafter, degradation of these intermediates occurred, presumably associated with the rupture of their furan ring (Figure 8b). In this regard, the best results were demonstrated by sample Bi_x_O_y_I_z_ (Figure 8c) which, after 8 h, provided an overall conversion value of 65%, with the FDCA yield reaching 15%. These results show the promise of sample Bi_x_O_y_I_z_ as a photocatalyst for deep oxidation of 5-HMF.

Table 1 presents data from the literature on the photocatalytic characteristics of bismuth oxyhalides with similar compositions previously obtained by various wet chemistry methods. It is important to note that accurately comparing catalyst activity based on the provided literature data is challenging, as the conditions of catalytic experiments in each report typically differ (e.g., the power and spectrum of irradiation sources, the concentrations of catalysts and analytes, etc.). In the future, we plan to compare the photocatalytic activity of similar bismuth oxyhalides obtained by laser and chemical methods under identical conditions to determine the relative efficacy of laser-generated nanomaterials compared to those produced by conventional approaches.

## 4. Conclusions

In this work, for the first time, the all-laser synthesis of bismuth oxyhalides (Bi_x_O_y_X_z_; X = Cl, Br, I) nanomaterials was demonstrated. This process involved the laser ablation of metallic bismuth in a medium of halogen-containing precursors (KX salts), followed by the additional laser treatment of the resulting colloid with a focused beam in plasma mode. The produced nanopowders predominantly comprised BiOCl, Bi_4_O_5_Br_2_, and Bi_4_O_5_I_2_ as their main phases (95% or more).

Photocatalytic tests demonstrated that the laser-produced bismuth oxyhalides were highly efficient catalysts for decomposing various organic compounds, including Rhodamine B, phenol, and tetracycline. Analysis of the absorption spectra of organic pollutants during photocatalysis revealed the destruction of strong cyclic aromatic structures (such as benzene rings) as a key feature of these novel nanocatalysts. Additionally, one of the nanomaterials, Bi_x_O_y_I_z_, exhibited excellent conversion and selectivity in the yield of target products, including deep oxidation to 2,5-furandicarboxylic acid, during the photooxidation of 5-hydroxymethylfurfural in aqueous solution.

The results suggest that bismuth oxyhalide nanomaterials synthesized by laser in water provide a promising foundation for multifunctional catalysts for the photoconversion of organic compounds. Future research will focus on the controlled laser synthesis of bismuth oxyhalides with varying stoichiometries and complex oxides, incorporating two or more halogen atoms simultaneously, as well as on preparing heterostructures based on these materials. Additionally, detailed studies of the photocatalytic mechanisms involving the newly developed nanocatalysts will be conducted, including assessments of the contributions of various active forms to the pathways of chemical transformations and their efficiencies.

## Figures and Tables

**Table 1:** Photocatalytic properties of bismuth oxyhalides reported in the literature.

Photocatalyst/ Method of Synthesis	Pollutant/Precursor	Experimental Conditions	Results	Ref
BiOCl/ hydrothermal	TC	Xe lamp 300 W, Catalyst: 10 mg, TC: 10 mg/L, 50 mL	Degradation 40% (120 min) *K* = 0.0037 min^−1^	[49]
BiOCl/ hydrolysis with UV light treatment	Rh B	Tungsten lamp 150 W, Catalyst: 20 mg, Rh B: 10 ppm, 50 mL	Dark sorption 50% (30 min) Degradation 100% (20 min)	[52]
BiOCl/ solvothermal	Rh B	Xe lamp 300 W (4.1 mW/cm^2^) with cut-off filter 400 nm Catalyst: 20 mg, Rh B: 20 mg/L, 100 mL	Degradation 99% (15 min) *K* = 0.272 min^−1^	[68]
BiOCl/ co-precipitation	Rh BTC	Xe lamp 300 W with filter 400 nm Catalyst: 20 mg, Rh B: 10 ppm, 50 mL TC: 10 ppm, 50 mL	Degradation Rh B 98% (60 min) Degradation TC 48% (120 min)	[69]
BiOCl/ co-precipitation	5-HMF	Xe lamp 300 W, Catalyst: 20 mg, 5-HMF 0.1 mM, 5 mL in acetonitrile	Conversion 78% (120 min) Selectivity 18% (DFF)	[66]
Bi_4_O_5_Br_2_/ solvothermal	TC	Xe lamp 1000 W with filter 420 nm Catalyst: 30 mg TC: 30 mg/L, 50 mL	Degradation 99% (120 min) *K* = 0.018 min^−1^	[70]
Bi_4_O_5_Br_2_/ solvothermal	Rh B	Xe lamp 300 W with filter 400 nm Catalyst: 25 mg Rh B: 0.0835 mM, 100 mL	Degradation 58% (25 min) *K* = 0.0065 min^−1^	[71]
Bi_4_O_5_Br_2_/ co-precipitation and calcination	Phen	Xe lamp 300 W with filter 400 nm Catalyst: 50 mg Phen: 10 mg/L, 100 mL	Degradation 80% (180 min)	[72]
Bi_4_O_5_I_2_/ co-precipitation	Rh B	Xe lamp 500 W with filter 420 nm Catalyst: 20 mg Rh B: 20 mg/L, 100 mL	Degradation 92.3% (120 min) *K* = 0.025 min^−1^	[73]
Bi_4_O_5_I_2_/ hydrothermal	Rh B	Xe lamp 300 W with filter 420 nm Catalyst: 20 mg Rh B: 20 mg/L, 100 mL	Degradation 92% (25 min) *K* = 0.08067 min^−1^	[74]
BiOCl/ laser synthesis	Rh B, TC, Phen	LED 375 nm, 50 mW optical power Catalyst: 15 mg Rh B: 5 µM, 30 mL TC: 50 µM, 30 mL Phen: 50 µM, 30 mL	Degradation Rh B: 100% (25 min) Dark sorption Rh B: 57% (60 min) Degradation TC: 30% (180 min) Degradation Phen: 51% (8 h)	This work
Bi_4_O_5_Br_2_/ laser synthesis			Degradation Rh B: 100% (30 min) Dark sorption Rh B: 63% (60 min) Degradation TC: 87% (180 min) Degradation Phen: 63% (8 h)	
Bi_4_O_5_I_2_/ laser synthesis			Degradation Rh B: 100% (120 min) Dark sorption Rh B: 75% (60 min) Degradation TC: 79% (180 min) Degradation Phen: 100% (180 min)	

## Data Availability

Dataset available on request from the authors.
